# Feasibility of quantification of the distribution of blood flow in the normal human fetal circulation using CMR: a cross-sectional study

**DOI:** 10.1186/1532-429X-14-79

**Published:** 2012-11-26

**Authors:** Mike Seed, Joshua F P van Amerom, Shi-Joon Yoo, Bahiyah Al Nafisi, Lars Grosse-Wortmann, Edgar Jaeggi, Michael S Jansz, Christopher K Macgowan

**Affiliations:** 1 Department of Paediatrics, Division of Paediatric Cardiology, University of Toronto, Hospital for Sick Children, 555 University Avenue, Toronto, ON M5G 1X8, Canada; 2 Department of Medical Biophysics, University of Toronto, Hospital for Sick Children, Toronto, Canada; 3 Department of Diagnostic Imaging, University of Toronto, Hospital for Sick Children, Toronto, Canada

**Keywords:** Regional blood flow, Magnetic resonance imaging, Circulation

## Abstract

**Background:**

We present the first phase contrast (PC) cardiovascular magnetic resonance (CMR) measurements of the distribution of blood flow in twelve late gestation human fetuses. These were obtained using a retrospective gating technique known as metric optimised gating (MOG).

**Methods:**

A validation experiment was performed in five adult volunteers where conventional cardiac gating was compared with MOG. Linear regression and Bland Altman plots were used to compare MOG with the gold standard of conventional gating. Measurements using MOG were then made in twelve normal fetuses at a median gestational age of 37 weeks (range 30–39 weeks). Flow was measured in the major fetal vessels and indexed to the fetal weight.

**Results:**

There was good correlation between the conventional gated and MOG measurements in the adult validation experiment (R=0.96). Mean flows in ml/min/kg with standard deviations in the major fetal vessels were as follows: combined ventricular output (CVO) 540±101, main pulmonary artery (MPA) 327±68, ascending aorta (AAo) 198±38, superior vena cava (SVC) 147±46, ductus arteriosus (DA) 220±39,pulmonary blood flow (PBF) 106±59,descending aorta (DAo) 273±85, umbilical vein (UV) 160±62, foramen ovale (FO)107±54. Results expressed as mean percentages of the CVO with standard deviations were as follows: MPA 60±4, AAo37±4, SVC 28±7, DA 41±8, PBF 19±10, DAo50±12, UV 30±9, FO 21±12.

**Conclusion:**

This study demonstrates how PC CMR with MOG is a feasible technique for measuring the distribution of the normal human fetal circulation in late pregnancy. Our preliminary results are in keeping with findings from previous experimental work in fetal lambs.

## Background

Galen (AD 129–200) first described the presence of the ductus arteriosus, foramen ovale and placenta in the human fetus. However, it was not until 1628 that William Harvey introduced the concept of a fetal circulation with the right and left heart ejecting in parallel and shunting at the foramen ovale and arterial duct bypassing the lungs [1]. In the second half of the twentieth century the distribution of the fetal circulation was studied in detail, initially by Dawes who made blood gas and flow measurements using plethysmography and intravascular flow probes in fetal lambs [2]. Rudolph and Heymann subsequently developed a technique for making more accurate measurements of fetal lamb blood flow under more physiological conditions using radioactive microspheres [3]. Our current understanding of the distribution of the fetal circulation is still based on these animal experiments, while subsequent ultrasound (US) studies have provided human data for comparison [4-8].

The measurement of blood flow distribution in the human fetal circulation could be useful for clinical obstetrics. The management of fetal distress [9], fetal growth restriction [10], fetal anemia [11] and fetal cardiac disease [12] is primarily guided by the US assessment of fetal hemodynamics. However, routine clinical practise does not usually include the measurement of blood flow by US. This is partly because velocity waveforms, which are more easily obtained by US, provide clinically useful information. However, the limited use of US derived flows is also due to the inherent inaccuracies of the technique. Errors arise from difficulties in measuring the vessel diameter, obtaining a suitable angle of insonation and the assumption of uniform flow velocity across the vessel lumen [13,14].

In recent years phase contrast cardiovascular magnetic resonance (PC CMR) has become a widely used clinical tool for blood flow quantification in post-natal cardiovascular disease [15]. This non-invasive technique avoids the use of ionising radiation and correlates well with the Fick technique, which employs oxygen consumption and blood gas measurements [16,17]. By contrast with US, which estimates flow based on a sampled zone within the vessel lumen, PC CMR integrates the velocities from voxels comprising the entire cross sectional area of the vessel being interrogated to calculate the vessel flow. CMR can be oriented in any plane, is not affected by poor windows and subjects the fetus to no known risk [18].

For adequate spatial and temporal resolution, cardiac triggering is generally used for phase contrast flow quantification. Unfortunately, real-time fetal ECG signals are not readily available for CMR due to electrical insulation of the fetus and contamination of the fetal ECG signal by the maternal ECG [19].

Using a gating technique known as metric optimised gating, which employs an image metric to retrospectively synchronise the CMR data instead of a conventional cardiac trigger [20],we made cine PC CMR measurements in twelve late gestation fetuses. The results of the flow measurements are presented. We include some preliminary observations regarding the distribution of the human fetal circulation. We also present the results of an adult volunteer experiment designed to test the accuracy of MOG, and some reproducibility and internal validation of the fetal flow measurements.

## Methods

### Metric optimized gating

The MOG technique achieves time-resolved cine PC MR flow measurements without direct cardiac gating. It uses an image metric to identify a heart rate, which, when used as a hypothetical trigger to synchronise the CMR data, gives the most coherent flow information. In this way it identifies what the heart rate was during the acquisition and synchronises the information from the scan accordingly.

To allow for complete reconstruction in time resolved MR, each segment is sampled at every cardiac phase. Typically, an ECG signal or a peripheral pulse monitor is used for cardiac gating, however neither of these are readily available for fetal imaging.

In the absence of a cardiac gating signal, each segment is acquired continuously for a period of time longer than the expected fetal heart rate. This oversampled data is retrospectively reconstructed using hypothetical cardiac triggers across the normal range of fetal heart rates. Using these hypothetical cardiac triggers, the data are grouped by cardiac phase and reconstructed to produce a time-series of PC MR images. An image metric, in this case entropy, is evaluated on the reconstructed images to determine the level of mis-gating artefact. To improve its specificity to fetal pulsations, metric values are calculated over a region surrounding the target fetal vessel and its accompanying artefact. This process is repeated and the heart rate iteratively adjusted. The accepted reconstruction is that which optimizes the image metric.

To allow for heart rate variation during the acquisition, a constant heart rate is assigned to each half of the total acquisition. These heart rates are then independently adjusted until the image metric is optimized. Increasing the number of segments improves the potential agreement between the true and hypothetical trigger positions at the expense of processing time.

### Validation study

This prospective study was approved by the institutional ethics review board. Written consent was obtained from all adult volunteers and pregnant mothers.

Five adult volunteers underwent imaging on a commercial 1.5T CMR system (Avanto, Siemens Medical Solutions, Erlangen, Germany). The volunteers had localisers performed to target both common carotid and internal jugular veins simultaneously, and were scanned using a head and neck coil. The volunteers were exercised using an CMR-compatible bicycle until they reached a steady heart rate within the normal fetal heart rate range (110–160 beats per minute) [21]. They continued to cycle to maintain a steady average heart rate while cine PC measurements of the carotid and jugular vessels were made using conventional pulse gating with a finger pulse monitor. These pulse-gated images were acquired at high temporal and spatial resolution to provide accurate reference flow values. Immediately after the pulse-gated scan, the measurements were repeated using MOG. The scan parameters for the MOG PC measurements reflect the need for shorter scan times when imaging fetal vessels, where movement artefacts degrade image quality. The parameters used were felt to represent a reasonable compromise, with shorter scan times achieved whilst maintaining adequate temporal resolution to capture flow curve characteristics and spatial resolution to resolve flow in the vessels being interrogated (target vessel diameters: 4–10 mm). The following scan parameters were used for the MOG PC acquisitions: VENC 150 cm/s, slice thickness 5mm, field of view 240 mm, phase field of view 100% + 33% phase oversampling, matrix size 192×192, voxel size 1.25×1.25×5 mm, echo time 2.92 ms, repetition time 6.55 ms, flip angle 20°, 1 average and 4 views per segment. A typical R-R interval of 520 ms resulted in a temporal resolution of 52.4 ms giving approximately 10 true cardiac phases, which were interpolated to 15 calculated phases. A typical scan time for each vessel was 34 seconds.

### Fetal study

Twelve pregnant mothers underwent MR imaging with the same CMR system used in the adult volunteer study. No sedation was used. All fetuses were singletons and had normal echocardiograms and obstetric findings. The median gestational age was 37 weeks with an age range of 30–39 weeks. Prior to moving the patient into the CMR scan room, the fetal heart rate was measured for 5 minutes using a CTG device (GE Corometric, Fairfield, Connecticut, USA). CMR data was obtained ensuring oversampling of the lowest fetal heart rate detected by CTG, for later reconstruction using MOG. The mother was made comfortable in a supine or lateral decubitus position in the magnet and a cardiac surface coil was placed over the maternal abdomen. The cardiac and spine coils were used in combination for imaging. An imaging protocol was then followed, starting with localisers, followed by a steady state free precession (SSFP) breath hold 3-dimensional acquisition of the whole fetus, and 3-plane static SSFP anatomical images through the fetal thorax as proposed by previous fetal CMR publications [22,23].

Cine MOG PC acquisitions were then performed with the imaging plane prescribed perpendicular to the main pulmonary artery (MPA), ascending aorta (AAo), superior vena cava (SVC), ductus arteriosus (DA), descending aorta at the diaphragm (DAo), and umbilical vein (UV) using the anatomical images to plan the prescriptions, as in post natal PC CMR. The intra-abdominal portion of the UV, proximal to the portal branches, was targeted to avoid complex flow behaviour. Measurements were attempted in the right and left pulmonary arteries (RPA & LPA) to assess pulmonary flow. Pulmonary blood flow (PBF) was also calculated indirectly from MPA minus DA flow. The correlation between these two measurements of pulmonary blood was used as an indicator of the accuracy of the MOG technique. The combined ventricular output (CVO) was calculated as the sum of the MPA and AAo plus 3% of this sum estimated for coronary flow based on previous lamb data [24]. Foramen ovale (FO) flow cannot be measured directly, however, since left ventricular output is made up of AAo and coronary flow and left ventricular venous filling is comprised of FO flow and PBF, it follows that FO flow can be calculated as the sum of AAo and coronary flows minus PBF. The velocity encoding range was tailored for the individual vessels with: 150 cm/s for the MPA, AAo, DA and DAo; 100 cm/s for the RPA, LPA and SVC; and 50 cm/s for the UV. In five subjects, each measurement was repeated to assess for reproducibility. The fetal scans were completed in 45 minutes per subject.

### Post processing

Using analysis software created in our laboratory (MATLAB, MathWorks, USA), the individual PC measurements were reconstructed using metric optimization according to our published technique [20]. Processing was performed in a semi-automated manner, with the user selecting a region of interest centred on a pulsatile vessel on the magnitude image from the PC scan. The software then performed the MOG analysis for the region of interest selected and produced a map showing the metric value for every combination of average heart rates during the first and second halves of the acquisition within a range of heart rates from 110–180 beats per minute. The software identified the combination of heart rates with the lowest metric value and the user confirmed its appropriateness visually from its position shown on the map. Having identified the proper heart rates, the reconstructed PC CMR images were exported to standard commercial cardiovascular post processing software (Q-flow 5.2, Medis Medical Imaging Systems, Leiden, Netherlands) for flow quantification with regions of interest drawn around the vessels of interest. Two radiologists independently contoured the fetal PCs to assess for inter-observer variation. The flows obtained by the more experienced cardiovascular radiologist were used for further analysis.

In order to calculate indexed flows, we adopted a modified version of an established CMR segmentation technique to estimate the fetal weight using commercially available software (Mimics, Materialise Group, Leuven, Belgium) [23,25,26]. A semi-automated tracing tool was used to define the interface between the high signal amniotic fluid and lower signal uterus on the 3 dimensional SSFP acquisition of the whole uterus. The fetus was then separated from the amniotic fluid with a signal intensity threshold tool. Finally, a cutting and filling tool which interpolated between sample slices finalised the dataset and allowed the segmentation software to calculate the fetal volume. The fetal weight was then estimated from the fetal volume using a conversion factor developed by Baker *et al.*based on fetal density: 0.12 + 1.031 ×fetal volume (ml) = MR weight (g) [25,26]. The total post processing time for a case was approximately 2 hours, including 30 minutes for the segmentation.

### Statistical methods

With the exception of the box and whisker plot, which uses medians and quartiles, all values in the text are expressed as means ± standard deviations. The adult and fetal flows in the reproducibility and inter-observer validation plots are the total values in milliliters per minute (ml/min) while the fetal flows have been indexed to the fetal weight and are given in milliliters per minute per kilogram (ml/min/kg). Fetal flows are also expressed as percentages of the CVO. For each vessel, flows measured between the subjects were normally distributed. Linear regression and Pearson’s correlation coefficient (R) were used to compare measurements of the same flows made with different techniques, the relationship between FO shunt and PBF, and the inter-observer agreement using MATLAB. Bland-Altman plots were also used to compare sequential measurements made in fetal vessels, the inter-observer variation for the same fetal flows, and the comparison of MOG and conventional gating in the adult validation experiment.

## Results

### Validation study

**Figure 1:**
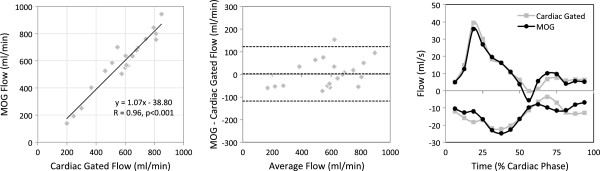
**Validation study of five adult volunteers comparing conventional vs MOG measurements of flow.** Measurements from the common carotid arteries and jugular veins made using conventional cardiac gated PC MR compared with the same measurements made by PC MR with MOG. The scatter plot shows agreement between the two methods while the Bland Altman plot reveals negligible bias. Flow curves measured in the right common carotid and right internal jugular vein by the two methods for one volunteer are shown.

Conventional pulse gated and MOG flow measurements were successfully made in the right and left common carotid arteries and jugular veins in all five subjects and are shown in Figure 1. The scatter plot shows the high level of agreement between the two techniques for both types of vessels (R=0.96, p<0.001). Agreement was consistent across a range of flows from 139 ml/min to 943 ml/min with no significant bias and a mean difference of 3.25±61.75 ml/min, as shown in the Bland Altman. The flow pattern was also reproduced well by the MOG analysis as shown by representative flow curves from a single carotid artery and jugular vein using both techniques. Heart rates during the volunteer studies ranged from 110–160 bpm.

### Fetal study

**Figure 2:**
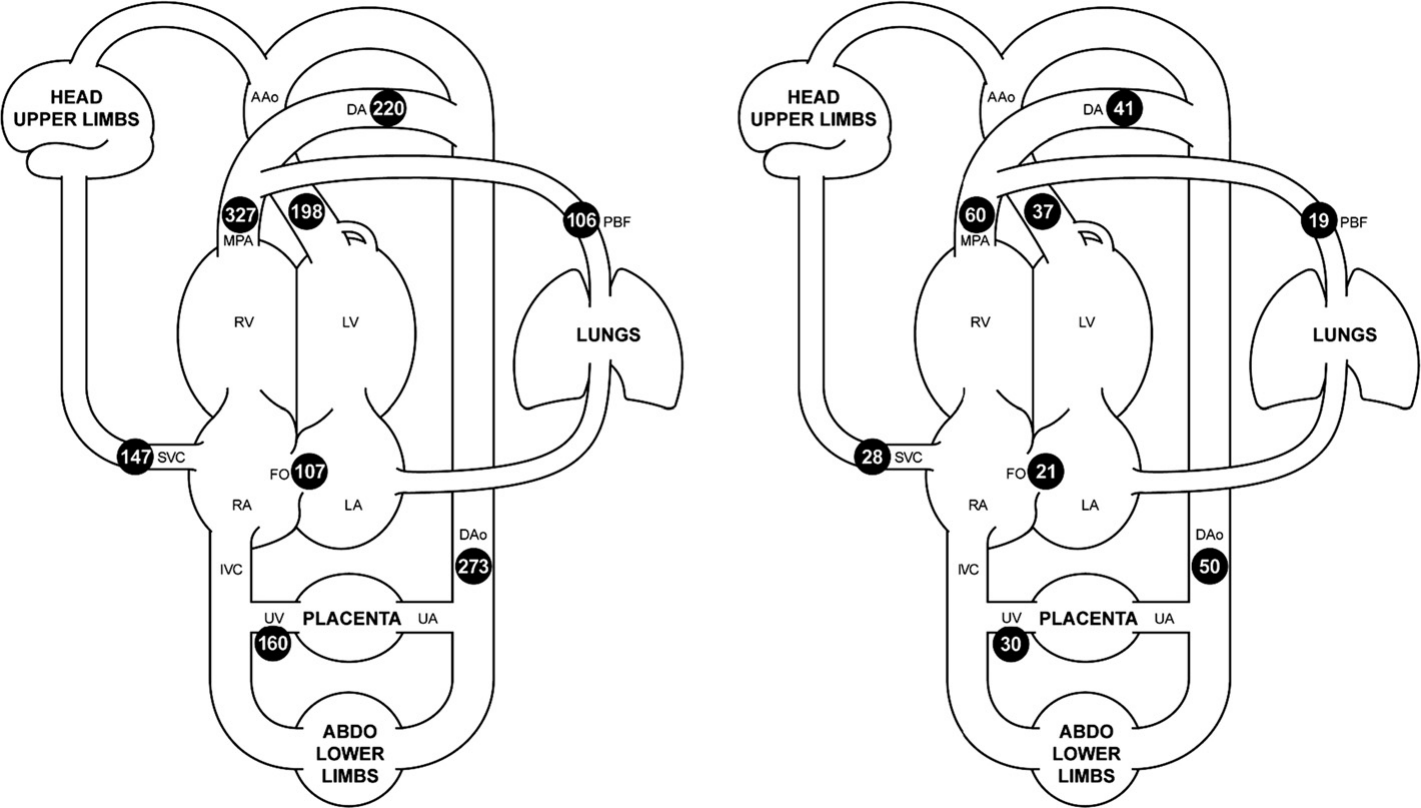
**Mean flows in the major vessels of the human fetal circulation by phase contrast MRI.** Mean flows in ml/kg/min (left) and proportions of the combined ventricular output (right) in the major vessels of the human fetal circulation by phase contrast MRI. Ascending aorta (AAo), main pulmonary artery (MPA), ductus arteriosus (DA), pulmonary blood flow (PBF), descending aorta (DAo), umbilical artery (UA), umbilical vein (UV), inferior vena cava (IVC), superior vena cava (SVC), right atrium (RA), foramen ovale (FO), left atrium (LA), right ventricle (RV), left ventricle (LV).

**Figure 3:**
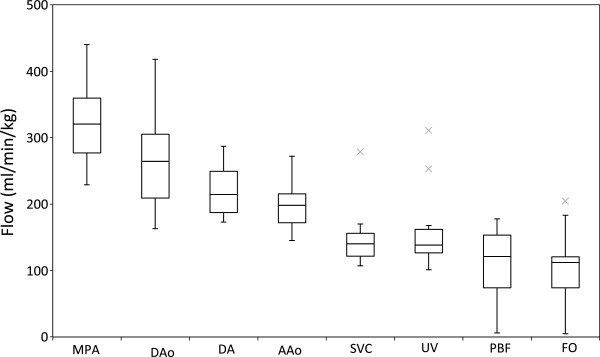
**Fetal flows measured in the major vessels by PC MR with MOG, indexed by weight.** Boxes indicate 1^st^ and 3^rd^ quartiles (Q1 and Q3, shown as lower and upper edges of the box) and 2^nd^ quartile (median, shown as band within the box). Upper and lower whiskers mark minimum and maximum values with the range Q1-1.5(Q3-Q1) to Q3+1.5(Q3-Q1). Outliers are marked with an X. MPA- main pulmonary artery, DAo- descending aorta, DA- ductus arteriosus, AAo- ascending aorta, SVC- superior vena cava, UV- umbilical vein, PBF- pulmonary blood flow, FO- foramen ovale.

Flows in all of the fetal vessels were possible in eight subjects. In the remaining four subjects, all of the flows were obtained with the exception of the branch pulmonary arteries. Failure to target the branch pulmonary arteries was due to poor slice prescription for these small vessels, which in turn was likely the result of fetal motion occurring in the interval between the localiser scans and the flow measurement being acquired. The flows from the fetal vessels are shown in Table 1 and Figures 2 and 3. An example of a phase contrast measurement made in the fetal descending aorta is shown in Additional file 1. Fetal weights were all above the 10^th^percentile for gestational age. We found variation in the CVO from 418 ml/min/kg to 733 ml/min/kg with a mean CVO of 540± 101 ml/min/kg. The relative contributions of the right and left ventricles were assessed based on the MPA flow versus the AAo plus estimated coronary blood flow. The right ventricle consistently provided 60±4% and left ventricle 40±4% of the CVO. The SVC blood flow was fairly consistent with a mean of 147±46 ml/min/kg or 28±7% of the CVO. The UV flow was reasonably constant at 160±62 ml/min/kg or 30±9% of the CVO. The right to left shunt across the FO was more variable at 107±54 ml/min/kg or 21±12% with a range of 1% to 44% of the CVO. The PBF, calculated as MPA minus DA, also varied with a mean of 106±59 ml/min/kg or 19±10% with a range 4% to 30% of the CVO.

**Table 1:** Fetal flows measured in the major vessels by PC MR with MOG, indexed by weight

**Absolute**	**Values**												
			**Measured**								**Calculated**		
**Subject**	**GA [weeks]**	**Weight[kg]**	**MPA [ml/min/kg]**	**DAo[ml/mn/kg]**	**DA[ml/min/kg]**	**AAo[ml/min/kg]**	**SVC[ml/min/kg]**	**UV[ml/min/kg]**	**RPA[ml/min/kg]**	**LPA[ml/min/kg]**	**PBF[ml/min/kg](MPA-DA)**	**FO[ml/min/kg](CVO-MPA-PBF)**	**CVO[ml/min/kg] (AAo+ MPA+3%)**
1	39	4.0	278	197	188	155	107	107	53	44	90	78	446
2	35	2.2	440	389	287	247	279	168			153	115	708
3	36	3.0	351	268	196	145	136	140			155	5	511
4	37	3.0	274	213	186	182	170	127	45	39	88	108	470
5	38	2.9	278	279	245	157	125	101	13	6	33	137	448
6	34	2.0	341	256	173	208	107	125	74	51	168	56	565
7	37	3.7	265	383	243	212	128	253	4	19	22	204	491
8	37	3.3	315	173	174	188	145	160	92	54	141	62	518
9	35	2.5	384	274	262	215	156	137			122	111	617
10	30	2.2	440	418	262	272	112	311			178	115	733
11	37	3.4	326	261	206	217	144	134	30	30	120	113	559
12	38	2.7	229	163	223	177	156	156	7	7	6	183	418
Mean	36	2.9	327	273	220	198	147	160			106	107	540
SD	2	0.6	68	85	39	38	46	62			59	54	101
Percentage of CVO													
			Measured								Calculated		
Subject			MPA[% of CVO]	DAo [% of CVO]	DA [% of CVO]	AAo [% of CVO]	SVC[% of CVO]	UV [% of CVO]	RPA[% of CVO]	LPA [% of CVO]	PBF [% of CVO](MPA-DA)	FO [% of CVO](CVO-MPA-PBF)	
1			62	44	42	35	24	24	12	10	20	17	
2			62	55	41	35	39	24			22	16	
3			69	52	38	28	27	27			30	1	
4			58	45	40	39	36	27	10	8	19	23	
5			62	62	55	35	28	23	3	1	7	31	
6			60	45	31	37	19	22	13	9	30	10	
7			54	78	49	43	26	51	1	4	4	42	
8			61	33	34	36	28	31	18	10	27	12	
9			62	44	42	35	25	22			20	18	
10			60	57	36	37	15	42			24	16	
11			58	47	37	39	26	24	5	5	21	20	
12			55	39	53	42	37	37	2	2	1	44	
Mean			60	50	41	37	28	30			19	21	
SD			4	12	8	4	7	9			10	12	

GA- gestational age, MPA- main pulmonary artery, DAo- descending aorta, DA- ductusarteriosus, AAo- ascending aorta, SVC- superior vena cava, UV- umbilical vein, RPA- right pulmonary artery, LPA- left pulmonary artery, PBF- pulmonary blood flow (MPA-DA), FO- foramen ovale, CVO- combined ventricular output.

**Figure 4:**
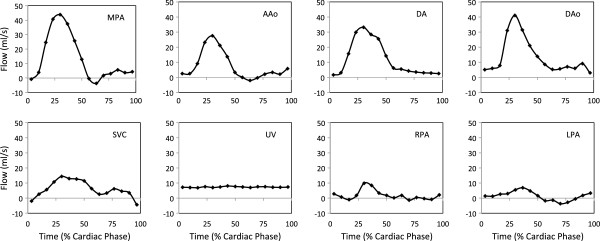
**Representative flow curves of the major fetal vessels, measured in one subject by PC MR with MOG.** MPA- main pulmonary artery, DAo- descending aorta, DA- ductus arteriosus, AAo- ascending aorta, SVC- superior vena cava, UV- umbilical vein, RPA- right pulmonary artery, LPA- left pulmonary artery, PBF- pulmonary blood flow, FO- foramen ovale, CVO- combined ventricular output.

**Figure 5:**
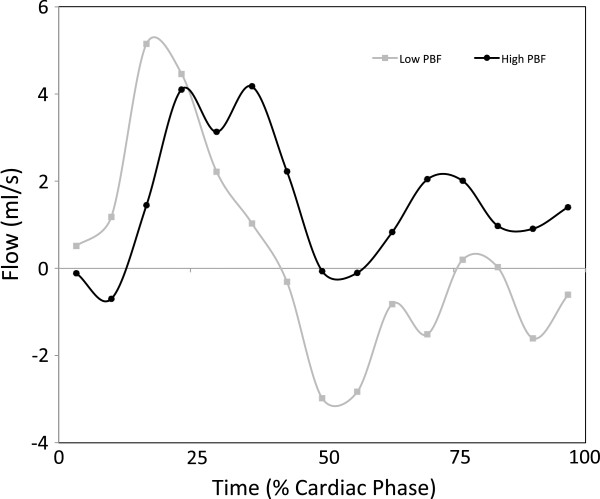
Flow curves from the left pulmonary artery in two subjects with high and low pulmonary blood flow.

A typical set of fetal flow curves obtained from subject number 5 is shown in Figure 4. The systolic forward flow in the MPA and AAo returns to baseline at end systole with a small amount of forward flow again noted in mid-diastole. DA flow has its systolic peak later in systole and continues into early diastole while the DAo flow has a more substantial and continuous diastolic component. The SVC has a biphasic flow pattern with systolic and diastolic peaks and a brief episode of flow reversal during atrial contraction. The branch pulmonary arterial flow patterns in this example show antegrade systolic flow with a trace of retrograde diastolic flow in the LPA. A continuous flow pattern is seen in the UV. Figure 5 shows the LPA flow pattern in fetuses 3 and 5. Fetus 3 had high PBF and fetus 5 had low PBF and the flow curves show contrasting antegrade and retrograde diastolic flow.

We investigated relationships between the different flows. No relationship between flow distribution and gestational age or fetal weight was identified. We found an inverse relationship with a strong correlation between PBF and right-to-left shunt across the FO (R=0.91, p=0.002, PBF = −0.84·FO + 170.47), where PBF was calculated as RPA + LPA to maintain independence between calculated flows.

### Fetal flow reproducibility, internal validation, and inter-observer variation

**Figure 6:**
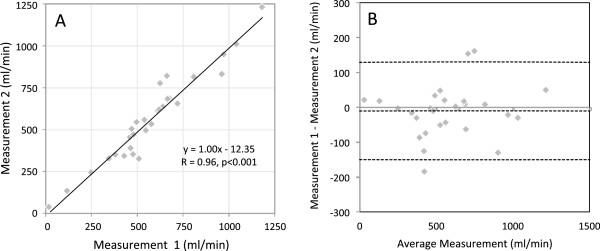
Reproducibility and bias of repeat MOG PC measurements of fetal flow in the major fetal vessels in five fetal subjects.

**Figure 7:**
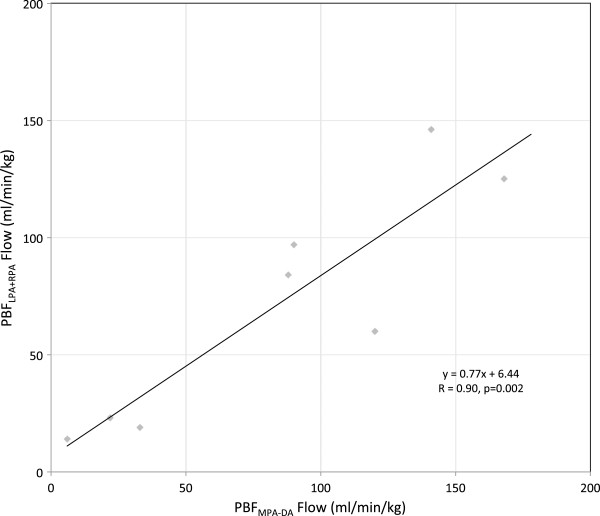
**Internal validation of fetal flows measured by PC MR with MOG.** Each data point compares two measures of PBF in the same subject; one the sum of LPA and RPA, the other the difference between MPA and DA.

**Figure 8:**
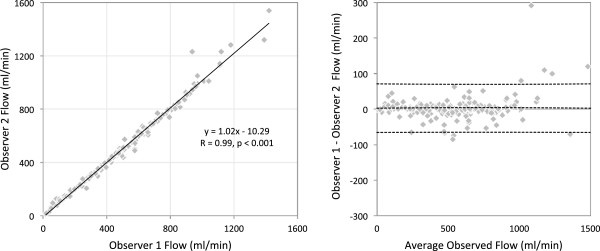
Agreement and bias between independent observers for all fetal flows across all twelve subjects measured by MOG PC MRI.

In five fetal subjects the flow in each vessel was measured twice. These flows, which have not been indexed to fetal weight in this case, are shown in the scatter plot and Bland Altman plot in Figure 6. A high level of reproducibility was found in the measurements across a range of flows from 116 to 1230 ml/min (R=0.96, p<0.001). In eight fetuses it was possible to measure pulmonary blood flow indirectly using MPA minus DA, and directly by making measurements in the RPA and LPA. Good agreement was found between these measurements, as shown in the scatter plot in Figure 7 (R=0.90, p=0.002). Inter-observer variation, shown in Figure 8, was low, with two observers in agreement (R=0.99, p<0.001) with a bias of 2.38 ± 34.86 ml/min across a range from 12 to 1540 ml/min.

## Discussion

### Accuracy of MOG PC CMR for fetal blood flow quantification

We present the preliminary results of measurement of the distribution of blood flow in the late gestation human fetus using PC CMR with MOG. We also include validation of our technique using an adult volunteer experiment designed to mimic fetal vessels whereby MOG could be compared with the gold standard using conventional cardiac triggering. The results of the validation experiment indicate a good correlation between flow measurements made in the neck vessels using conventional pulse gating and MOG. The small differences between the measurements made using conventional gating and MOG may at least partly be due to real changes in flow occurring between the times of acquisition of the two measurements. Such differences were minimised by making the MOG measurement immediately after the pulse-gated measurement. The flows were comparable to fetal flows in terms of vessel size (4-10mm diameter) and flow (139–943 ml/min). Correct modeling of fetal vessels is important because the accuracy of PC measurements depends on the size of the vessel and spatial resolution afforded by the scan parameters. The spatial resolution for the volunteer MOG measurements was the same as the fetal scan parameters and the number of pixels per vessel diameter was greater than 3 and up to 8, which has been shown to be sufficient for accurate flow quantification [27]. Parallel-imaging techniques were not incorporated into the MOG reconstruction at the time of this study. However, parallel imaging will allow further improvement in spatial resolution without a corresponding increase in scan time. As well as matching the size and flows in fetal vessels in our adult validation, we also attempted to reproduce the fetal heart rate during the acquisitions. The measurements were therefore made after the subject had been cycling for 10 minutes on an CMR compatible bicycle, with the subject continuing to cycle at a steady rate during the acquisition to maintain a heart rate in the normal fetal range between 110 and 160 bpm. By matching the adult volunteer heart rates to fetal heart rates, we ensured a similar temporal resolution was achieved in the fetal and adult scans.

As MOG is a retrospective technique that uses modelled heart rates, a further possible source of inaccuracy in the flow measurements is heart rate variability. The pattern and range of heart rate variation is likely to be different between the fetal and adult scans. However, the normal fetus has less beat-to-beat variation in heart rate than an exercising adult [28], and is therefore better suited to measurement with MOG [20]. In summary, we hypothesise that the fetal measurements using MOG were at least as accurate as the measurements obtained in adults with similarly sized vessels, the same heart rate range but with greater inter-beat variability.

### Fetal flow results

Previous attempts to measure blood flow in the fetal circulation include those made by Rudolph in the fetal lamb using radioactive microspheres [3,24]. Access to the fetal vasculature was obtained after externalising the fetus. The fetus could then be returned to the uterus and microspheres introduced into the circulation. The microspheres were trapped in the capillaries of the end organs supplied by the fetal circulation where their activity could be measured, thus quantifying perfusion of the organ. Flow in fetal lamb vessels was also measured using ultrasound flow probes placed around the vessels [24]. The reports of this work helped to establish the principle of a combined ventricular output in the fetus, in which the right ventricle is dominant, and also presented mean flows and percentages of combined ventricular output for the pulmonary, cerebral and placental circulation as well as the magnitude of the shunts across the foramen ovale and ductus arteriosus. These lamb measurements were used to estimate the distribution of the human fetal circulation, with pulmonary and cerebral blood flow thought to be higher and placental blood flow lower in the human because of developmental differences between the species.

Several other investigators have published measurements of the human fetal circulation made non-invasively by ultrasound [4-8]. Ultrasound-derived fetal blood flow measurements are made using the product of the vessel area and time velocity integral of a Doppler sample of the vessel flow. The results of our CMR measurements are similar to these ultrasound measurements, and to the estimates based on lamb experiments [24]. The flow curves have similar patterns to Doppler ultrasound velocity patterns for each of the vessels shown in Figure 4 [12]. In keeping with Rudolph’s estimates and the majority of the ultrasound papers, we have found higher average pulmonary blood flow in the human compared with the lamb. We have also found a lower umbilical venous flow of approximately 160 ml/min/kg, which is in agreement with several ultrasound estimates [29,30]. The mean combined ventricular output by CMR of 540 ml/kg/min is higher than the majority of the ultrasound studies, although it is similar to one ultrasound study [5] and to the fetal lamb measurements [24]. A consistent finding between different studies is the considerable variation in the CVO between fetuses, as was the case in our study. Variation in the CVO in the same individual relating to different levels of fetal activity has also been observed in lamb studies, and this may be the explanation for the high flows seen in subject 2 (Table 1) [24].

One potential source of error in our indexed measurements was inaccuracy of the fetal weight estimation. However, CMR segmentation has been shown to be more accurate than ultrasound measurements of fetal weight, with good correlation (R = 0.95) and a mean error of 129 grams between CMR segmentation performed within 3 hours of delivery and actual birth weight [25]. The 3D SSFP we used for the segmentation has higher spatial resolution and a shorter scan time than the single-shot fast spin echo acquisition used in the above validation, and is therefore likely to be even more accurate [23].

Whilst previous authors have included normal ranges for their measurements, only Rasenan *et al.* explore the relationship between the foramen ovale shunt and pulmonary blood flow [7]. In our study a rather consistent ascending aortic flow was found in all fetuses. The venous return to the left ventricle is supplied by two possible sources in the fetus, pulmonary venous return and foramen ovale shunt. We noted a strong inverse relationship between these two competing sources. In fetuses with high pulmonary blood flow, there was low foramen ovale flow, while larger foramen ovale shunts were found in fetuses with low pulmonary blood flow. In Rasenen’s study there was a shift from filling of the left heart by foramen ovale shunt towards filling via the pulmonary circulation as the fetus matured. Our measurements were limited to third trimester subjects. However, within this more limited gestational age range, the eight-fold difference in pulmonary blood flow and wide range of right-to-left foramen ovale shunts showed no relationship to fetal maturity.

Inaccuracies in PC CMR are more likely in small vessels with low flows and the measurement of pulmonary blood flow in the fetus represents the limit of what is currently possible with this technique. This is demonstrated by our failure to make these measurements in four of twelve subjects. Subtraction of one flow from another made at a different time, as in the quantification of pulmonary flow from MPA and DA here is also likely to lead to errors. However, the wide range of normal pulmonary blood flow measured by both techniques as shown in Figure 7, and the variation in the branch pulmonary arterial flow pattern seen in Figure 5 suggest there is a wide physiological range of pulmonary vascular resistance in the normal fetus. The mechanisms and consequences involved are likely to relate to streaming patterns and oxygen handling by the fetal circulation, although further discussion of this subject is beyond the scope of this report.

### Study limitations

A major limitation of the current study is the lack of a gold standard for the fetal studies. However, the validation study includes the accepted gold standard of conventionally gated PC CMR flow measurement, albeit of carotid arterial and jugular venous flows in adult volunteers where high signal to noise ratio, appropriate slice location and freedom from movement artifacts are more likely to be achievable than in the measurements of fetal vascular flow. PC CMR with MOG should be compared with ultrasound for normal human fetal flows, but this was not attempted in this study, which was primarily designed to investigate the feasibility of the new technique. Another limitation is the small number of fetal subjects. The novelty of this approach warrants the reporting of this initial sample, and further studies with larger numbers of subjects will establish normal ranges for fetal flows measured with CMR, ideally over a range of gestational ages. Our initial experience suggests this technique is best suited to mature fetuses, when there is an adequate signal returned from the fetal vessels and when fetal motion is less vigorous. However, fetal imaging aims to identify fetal abnormalities as early in the pregnancy as possible, when more treatment options may be available. A further limitation of our study is that we have not as yet examined whether the technique is feasible when abnormalities of the fetal cardiovascular system are present. However, we do not anticipate major problems with applying the technique to similarly sized fetuses with hemodynamic disturbances. Although the current post processing time makes this technique labour intensive, the time required to analyse a study is equivalent to post-natal cardiovascular CMR studies with established clinical indications.

## Conclusions

This paper shows that measurement of human fetal blood flow distribution is possible by PC CMR with MOG. An in-vivo adult volunteer experiment, designed to mimic fetal vessel flows, demonstrated the agreement between conventionally gated PC CMR and MOG measurements of flow. The reliability of the technique was further supported by good reproducibility and internal validation of fetal flows. The preliminary findings using this technique were in keeping with previously published estimates of flow distribution in the fetal circulation. However, the measurements of foramen ovale and pulmonary blood flow suggest that reciprocal variation in these flows may be present in normal human fetal circulatory physiology, allowing for some ‘play’ in the source of the venous return to the left ventricle.

We believe that using standard clinical equipment, PC CMR with MOG offers the potential to re-examine normal and abnormal human fetal circulatory physiology. Possible future applications in the fetus might include assessment of the redistribution of blood flow associated with fetal distress, which might be useful to inform decision-making regarding early delivery of the fetus. Another promising application is the assessment of congenital heart malformations, in which fetal hemodynamics including pulmonary and cerebral vascular resistance and flow redistribution can play a role in the postnatal presentation and natural history of the disease.

## Abbreviations

PC: Phase Contrast; MOG: Metric Optimised Gating; CVO: Combined Ventricular Output; MPA: Main Pulmonary Artery; AAo: Ascending Aorta; SVC: Superior Vena Cava; DAo: Descending Aorta; DA: Ductus Arteriosus; PBF: Pulmonary Blood Flow; UV: Umblical Vein; FO: Foramen Ovale; RPA: Right Pulmonary Artery; LPA: Left Pulmonary Artery; US: Ultrasound; CTG: Cardiotocograph; SSFP: Steady State Free Precession.

## Competing interests

The authors declare they have no competing interests.

## Authors' contributions

MS designed the study, performed the examination and drafted the manuscript. JFPVA contributed to the development of MOG, analysed the data and produced the figures. SJY concieved of the basic principle for MOG and helped develop the imaging steps. BAN was one of the radiologists in the inter-observer variability assessment. LGW contributed to the development of the fetal CMR protocol. EJ provided guidance on the fetal circulation, helped with the analysis and manuscript. MSJ achieved MOG. CKM was the senior scientist responsible for MOG, supervised MSJ and JFPVA and helped to draft the manuscript. All authors read and approved the final manuscript.

## Supplementary Material

Additional file 1 Fetal Descending aortic PC MRI measurement with metric optimised gating. Click here for file
